# Enhancing the Outcomes of Temporalis Fascia Tympanoplasty Using Autologous Platelet-Rich Plasma and Gel: A Randomized Controlled Trial

**DOI:** 10.3390/jpm15060233

**Published:** 2025-06-04

**Authors:** Nejc Steiner, Domen Vozel, Nina Bozanic Urbancic, Kaja Troha, Andraz Lazar, Veronika Kralj-Iglic, Saba Battelino

**Affiliations:** 1 Otorhinolaryngology Department, UMC Ljubljana, Zaloska cesta 2, 1000 Ljubljana, Slovenia; 2 Medical Faculty, University of Ljubljana, Vrazov Trg 2, 1000 Ljubljana, Slovenia; 3 Otorhinolaryngology Department, Wythenshawe Hospital, Manchester University National Health Service Foundation Trust, Manchester M239LT, UK; 4 Department Psychological Methodology, Faculty of Arts, University of Ljubljana, Askarceva 2, 1000 Ljubljana, Slovenia; 5 Biotechnical Faculty, University of Ljubljana, Jamnikarjeva 101, 1000 Ljubljana, Slovenia

**Keywords:** tympanoplasty, platelet-rich plasma (PRP), platelet-rich gel (PRG), extracellular vesicles, tympanic membrane perforation, cartilage graft, hearing improvement, quality of life, randomized controlled trial, otologic surgery

## Abstract

**Objectives:** This study aimed to investigate the impact of platelet-rich plasma (PRP) and platelet-rich gel (PRG) on tympanic membrane closure rates, hearing improvement, and quality of life following tympanoplasty. **Methods:** Seventy-two patients with chronic tympanic membrane perforations were enrolled in a double-blinded, randomized controlled trial at a single tertiary referral center. All patients underwent tympanoplasty using a temporalis fascia graft and were randomly assigned to one of two groups: one group received standard tympanoplasty alone, while the other received intraoperative application of autologous PRP and PRG, in addition to the standard procedure. **Results:** The PRP group demonstrated a significantly higher rate of complete tympanic membrane closure compared to the control group (32/36; 88.9% vs. 24/36; 66.7%; *p* < 0.05). Bone conduction hearing remained unchanged in both groups, while air conduction hearing improved significantly from pre- to post-treatment in each group. However, the difference in air conduction improvement between the PRP group and the control group was not statistically significant (PRP group: Mdn = −8.25; control group: Mdn = −12.20; U = 618; z = −0.54; *p* = 0.30). Quality of life improved in both the PRP and control groups; however, the difference between the groups was not statistically significant (PRP group: 10.44 ± 10.46; control group: 10.47 ± 8.22; 95% CI [−4.45; 4.40]; *t*(66) = −0.01; *p* = 0.16). **Conclusions:** Our findings suggest that intraoperative application of autologous PRP and PRG may improve tympanoplasty outcomes, particularly in cases with lower expected success rates or when performing minimally invasive transcanal procedures under local anesthesia. However, variability in PRP preparation, application methods, and graft materials across studies limits direct comparisons. Standardized protocols and further controlled studies are necessary to clarify PRP’s clinical value in tympanoplasty.

## 1. Introduction

Tympanoplasty is a commonly performed surgical procedure with reported success rates in the literature ranging from 75% to 98% [1,2]. Despite these favorable outcomes, certain patient populations experience significantly lower rates of tympanic membrane healing. The success rate of tympanoplasty reported in the literature drops to 64.7% in cases where the perforation exceeds 50% of the total tympanic membrane area, 63.3% in patients with myringosclerosis, and 64.1% when less than three months have elapsed since the onset of middle ear discharge [3]. These findings underscore the challenges faced in achieving optimal healing in complex cases.

Various types of grafts can be used in tympanoplasty, including autologous materials such as temporalis fascia, fat graft, perichondrium, and cartilage, each offering distinct structural and healing properties suited to different clinical scenarios [4,5].

The regenerative capacity of the tympanic membrane plays a crucial role in the success of tympanoplasty, yet this intrinsic healing ability cannot be directly modified through surgical intervention alone. Consequently, despite high overall success rates, the proportion of patients achieving complete tympanic membrane closure remains suboptimal in certain scenarios. To address these challenges, it is essential to explore adjunctive therapies that may enhance surgical outcomes. One such promising intervention is the use of platelet-rich blood derivatives, including platelet-rich plasma (PRP) and platelet-rich gel (PRG), which have the potential to improve tympanic membrane healing.

PRP has been shown in various studies to accelerate and enhance the healing of damaged tissues [6,7,8,9,10,11] and to reduce the likelihood of postoperative inflammation [12]. It also contains extracellular vesicles, which, along with platelets, are the primary mediators of PRP’s regenerative effects [9,13].

This study seeks to provide new insights into the potential role of PRP and PRG in enhancing tympanoplasty outcomes, particularly in challenging cases where traditional surgical approaches may fall short.

## 2. Materials and Methods

This monocentric, prospective, double-blinded, parallel, evenly randomized, and controlled study received approval from the National Medical Ethics Committee on 13 January 2021 (0120-498/2020-3) and was conducted in accordance with the Helsinki Declaration [14]. It was registered on ClinicalTrials.gov (ID: NCT04761562, available online: https://clinicaltrials.gov/study/NCT04761562?term=steiner%20nejc&rank=1; accessed on 27 May 2025).

Prior to the study’s initiation, the required sample size was calculated to ensure sufficient statistical power (80%) for a dichotomous endpoint comparison between two independent samples. Based on variability in published success rates, a 75% success rate was assumed for the control group and a 98% success rate for the PRP group, the latter representing the highest rate reported in the literature [1,2]. To achieve 80% power, with an alpha significance level of 0.05 and a beta error rate of 0.20, the minimum required sample size was determined to be 66 subjects, with 33 participants allocated to each group.

### 2.1. Recruitment

Patients were recruited from 1 May 2021 to 15 February 2024 at the ENT Department, University Medical Centre Ljubljana, Slovenia. Age restrictions were not applied. Inclusion and exclusion criteria are detailed in Table A1 in the Appendix A.

### 2.2. Intervention Allocation

Patients were randomized 1:1 into two groups using a telephone-based system and a pre-generated sequence from WinPepi software (version 11.0) [15]. Group A was the control group, and Group B received PRP.

**Figure 1:**
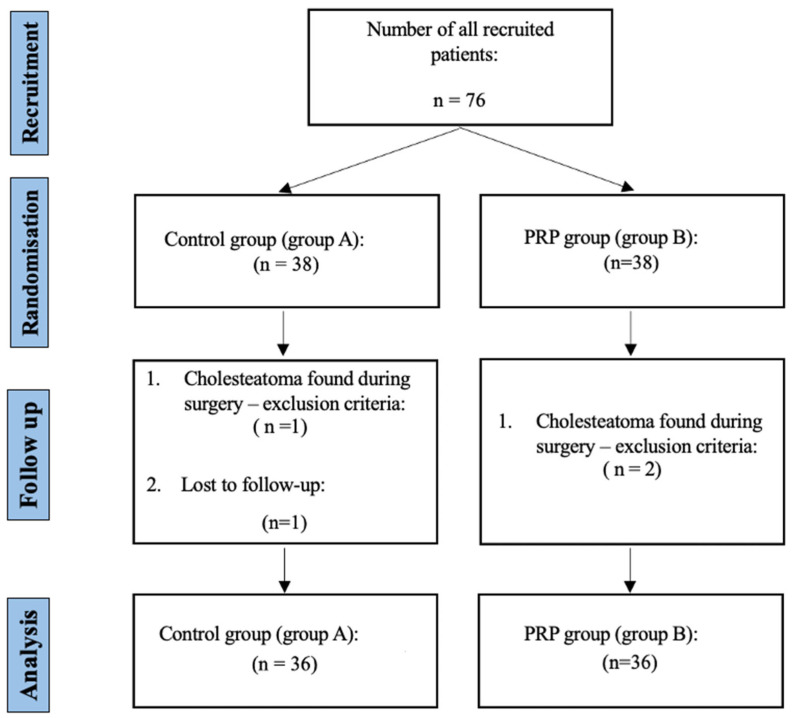
Study flowchart following the CONSORT guidelines [16]. Legend: PRP—platelet-rich plasma, and *n*—number of patients.

Seventy-six patients were recruited. During the surgical procedure, cholesteatoma, which is one of the exclusion criteria, was discovered in one patient from the control group and two patients from the PRP group. Therefore, these patients were excluded from the study. One patient from the control group did not attend the postoperative follow-up appointment and was thus excluded from the trial. In the final analysis, 72 patients were included, 36 from each group (Figure 1).

### 2.3. Preoperative Evaluation

Preoperative evaluation comprised a thorough medical history, detailed physical examination, extended high-frequency pure tone audiometry (up to 16 kHz), vestibular assessment including video head impulse testing (vHIT), and air caloric testing, as well as photographic documentation of the tympanic membrane perforation.

### 2.4. Surgical Treatment

**Figure 2:**
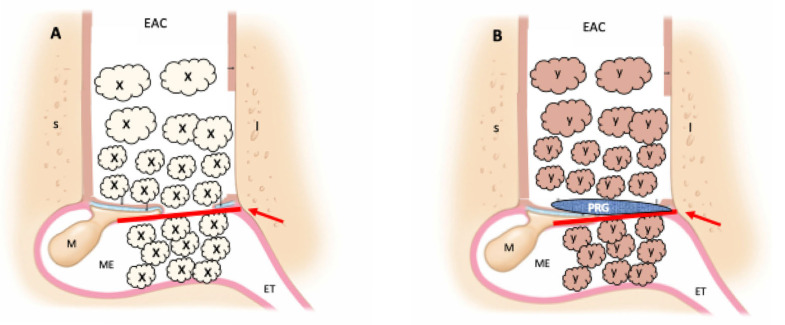
Surgical treatment. (**A**) Control group (classic tympanoplasty); (**B**) PRP group (tympanoplasty with addition of platelet-rich plasma and platelet-rich gel); red arrow—temporalis fascia; X—atelocollagen sponge with ciprofloxacin solution; Y—atelocollagen sponge with platelet-rich plasma and ciprofloxacin solution; PRG—platelet-rich gel; S—superior external ear canal wall; I—inferior external ear canal wall; EAC—external ear canal; ET—eustachian tube; M—malleus; ME—middle ear.

The surgical treatment for each group is illustrated in Figure 2.

#### 2.4.1. Surgical Management of the Control Group (Group A)

In the control group, either endaural or postauricular approaches were utilized. The edges of the perforation were refreshed, and the tympano-meatal flap was elevated. Temporalis muscle fascia was then placed underneath the flap. Additionally, an atelocollagen sponge (Gelfoam^®^, Pfizer, New York, NY, USA), saturated with 1 mL of ciprofloxacin solution (Ciloxan^®^, Alcon Laboratories, Geneva, Switzerland, 3 mg/mL), was applied beneath the graft and on the lateral surface of the reconstructed eardrum.

#### 2.4.2. Surgical Management of the PRP Group (Group B)

Patients treated with PRP underwent a surgical procedure similar to that of the control group, with the following additional steps:

- In addition to the 1 mL of ciprofloxacin solution (3 mg/mL), 1 mL of PRP was applied to numerous pieces of atelocollagen sponge. This preparation was then inserted into the middle ear cavity to provide medial support for the graft.
- PRG was applied to the lateral surface of the reconstructed eardrum.
- Finally, the external auditory canal was packed with the remaining pieces of atelocollagen sponge soaked in PRP and ciprofloxacin solution.

#### 2.4.3. Preparation of PRP and PRG

The PRP and PRG were prepared using a 2-step preparation protocol outlined by Vozel et al. (2020) [9]:

- Blood Collection: Nine milliliters of venous blood was drawn into two four and a half milliliters test tubes containing sodium citrate (9 NC sodium citrate 0.105 M, BD Vacutainer, Becton Dickinson, Franklin Lakes, NJ, USA).
- First Centrifugation: Conducted at 300× *g* for 5 min at 18 °C, resulting in the blood separating into three distinct layers (plasma, buffy coat and red blood cells).
  ○ Supernatant (plasma and buffy coat) was carefully removed from the tube and transferred into a new sterile, empty plastic tube.
- Second Centrifugation: Plastic tube with supernatant was centrifuged at 700× *g* for 17 min at 18 °C, resulting in the sedimentation of platelets and plasma at the bottom of the tube.
  ○ The upper half of centrifugate was removed and discarded, while the bottom, representing PRP, was collected. A total of 1 mL of PRP was used to prepare PRG, and the remainder was applied to atelocollagen sponge.
- PRG preparation: PRP was exogenously activated by mixing 1 mL of PRP with 10 μL of 1 M CaCl_2_ (1:100 ratio to PRP) and 0.2 mL of autologous serum (1:5 ratio to PRP).
  ○ Autologous serum is prepared by centrifuging whole blood in a 4 mL plastic test tube without anticoagulant (Z Serum, Vacutube, LT Burnik, Komenda, Slovenia) at 1260× *g* for 10 min at 18 °C. The resulting supernatant, representing the autologous serum, is then carefully collected.

### 2.5. Outcome Measures

#### 2.5.1. Primary Outcome Measure

The primary outcome of this study was the rate of tympanic membrane closure after tympanoplasty.

The primary outcome measure was individually assessed before and after tympanoplasty using high-resolution photographic documentation with an iPhone X paired with a 30-degree endoscope and iPhone X adapter (endoscope-i©), supported by the e-i Pro© application (version 2.2.6). To achieve patient-specific evaluation, the size of each eardrum perforation was quantified as a percentage of the individual’s tympanic membrane area using Adobe Photoshop© (version 22.1.0). Two independent reviewers, blinded to patient identity and treatment allocation, analyzed the images by precisely outlining both the perforated area and the entire tympanic membrane to obtain pixel counts. These personalized perforation percentages were calculated based on individual pixel ratios, and the final value for each patient was derived by averaging the two independent assessments. This approach enabled objective, reproducible, and individual-level analysis of surgical outcomes, consistent with the principles of personalized medicine.

#### 2.5.2. Secondary Outcome Measures

Secondary outcomes included hearing improvement and quality of life after PRP treatment. Hearing improvement was assessed using PTA before and after surgery, with the pure tone average calculated as the mean of hearing thresholds at 0.5 kHz, 1 kHz, 2 kHz, and 4 kHz. Quality of life was assessed using the COMQ-12, with higher scores reflecting a greater negative impact on quality of life. The maximum possible score was 60 points. To maintain accuracy and reliability, patients under 15 years of age were excluded from completing the questionnaire.

### 2.6. Postoperative Period

Patients underwent routine postoperative follow-up visits at 6 weeks and 6 months following surgery. At the 6-week follow-up, ear packing was removed.

Outcomes were assessed at the 6-month follow-up using the primary and secondary measures defined at the start of the study. Tympanic membranes were re-photographed, and PTA and vestibular assessment (vHIT, caloric testing) were repeated. Patients also completed the COMQ-12 questionnaire once more.

### 2.7. Adverse Event Monitoring and Assessment

Adverse effects were systematically monitored and recorded throughout the study by closely observing patients during follow-up visits, documenting any reported symptoms, complications, or unexpected outcomes and categorizing them based on severity and potential relation to the intervention.

### 2.8. Statistical Analysis

For data recording and editing, Microsoft Excel (versions 16.9.0–16.36) was utilized. Statistical analysis was conducted by a specialist statistician using R software, RStudio interface (version 4.1.2;). Statistical significance between groups was defined as a probability of rejecting the null hypothesis exceeding 95% (*p* < 0.05).

## 3. Results

The analysis of patient demographics (summarized in Table 1) revealed a similar gender distribution between the groups, with 19 males and 17 females in the PRP group compared to 18 males and 18 females in the control group (*p* = 1.000). The mean age of the patients was significantly higher in the PRP group (40.69 ± 18.27 years) than in the control group (32.42 ± 21.77 years, *p* = 0.045). The median size of tympanic membrane perforation was comparable between the groups, at 31% in the PRP group and 25.5% in the control group (*p* = 1.000).

We analyzed the rate of complete tympanic membrane closure in both the PRP and control groups (summarized in Table 2). The PRP group demonstrated a statistically significant higher healing rate compared to the control group (32/36; 88.9% vs. 24/36; 66.7%; *p* < 0.05 (Fisher’s exact test—the frequency count in one of the conditions was less than 5)).

There was no change in bone conduction hearing pre- and post-treatment in any of the groups (control group: preoperative Mdn = 10.35 dB, postoperative Mdn = 10.35 dB; *W* = 31.5; *z* = 0.21; *p* = 0.58 (Fisher’s exact test—the frequency count in one of the conditions was less than 5); PRP group: preoperative Mdn = 13.21 dB, postoperative Mdn = 13.57 dB; *W* = 97; *z* = −1.41; *p* = 0.08^2^).

Both the treatment and control groups showed a statistically significant improvement in air conduction hearing from pre- to post-treatment (control group: preoperative Mdn = 32.50 dB, postoperative Mdn = 18.25 dB; *W* = 1; *z* = −3.89; *p* < 0.05^2^; PRP group: preoperative Mdn = 39.75 dB, postoperative Mdn = 24.50 dB; *W* = 41; *z* = −3.63; *p* < 0.05 (Mann–Whitney *U* test)). However, the difference in the improvement of air conduction hearing between the treatment and control groups was not statistically significant (PRP group Mdn = −8.25 dB, control group Mdn = −12.20 dB; U = 618; z = −0.54; *p* = 0.30 (Fisher’s exact test—the frequency count in one of the conditions was less than 5)).

The COMQ-12 questionnaire demonstrated an improvement in quality of life from pre- to post-treatment within each treatment group. However, the improvement in quality of life was not statistically significantly greater in the PRP group (M = 10.44; SD = 10.46) compared to the control group (M = 10.47; SD = 8.22; 95% CI [−4.45; 4.40]; *t*(66) = −0.01; *p* = 0.16 (Fisher’s exact test—the frequency count in one of the conditions was less than 5)).

**Figure 3:**
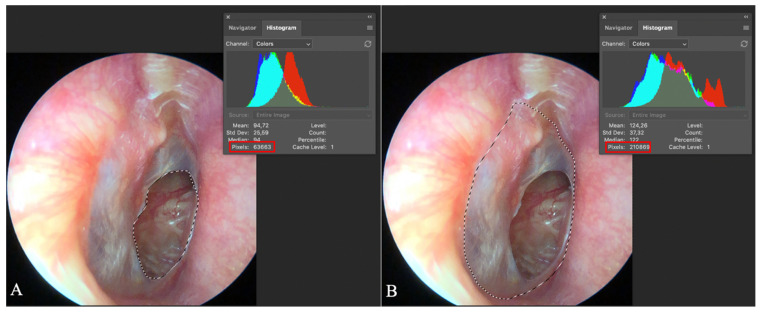
Evaluation of tympanic membrane perforation size. Adobe Photoshop© screen capture illustrates two marked areas: (**A**) the perforated region of the tympanic membrane circled, with the pixel count (63,663) displayed in the red quadrant; (**B**) the entire eardrum circled, with its pixel count (210,869) shown in the red quadrant. The percentage of perforation is calculated by dividing the number of perforation pixels by the total tympanic membrane pixels, resulting in a perforation percentage of 30.19% in this case.

Logistic regression analysis was used to evaluate the relationship between perforation size and the likelihood of successful healing while adjusting for potential confounding factors (Figure 3). For each perforation size, we calculated the probability of tympanic membrane healing.

Vestibular assessment conducted preoperatively and postoperatively yielded normal results. No adverse events related to platelet-rich plasma (PRP) or platelet-rich gel (PRG) were observed.

## 4. Discussion

The success rates for the surgical treatment of chronic non-cholesteatomatous otitis media, defined as complete tympanic membrane healing, range from 75% to 98% in adults, as reported in the literature [1,17,18]. In children, these rates show greater variability, spanning from 35% to 94% [19]. Factors such as patient age under sixteen, perforation size, the condition of the contralateral ear, and the surgeon’s experience significantly influence postoperative tympanic membrane healing. Additionally, the intrinsic regenerative potential of the tympanic membrane plays a pivotal role in the healing process, an aspect that surgical techniques alone cannot enhance [20]. Based on our findings, PRP and PRG could represent a promising option to promote and accelerate tympanic membrane healing, particularly in challenging cases. Extracellular vesicles in these preparations play a key role in promoting collagen production, which reduces perforation size, accelerates epithelialization, and enhances dermal angiogenesis [21]. During tympanoplasty, PRP and PRG stimulate collagen formation, a critical factor since larger tympanic perforations tend to have reduced collagen, necessitating additional collagen synthesis for effective closure [22].

**Figure 4:**
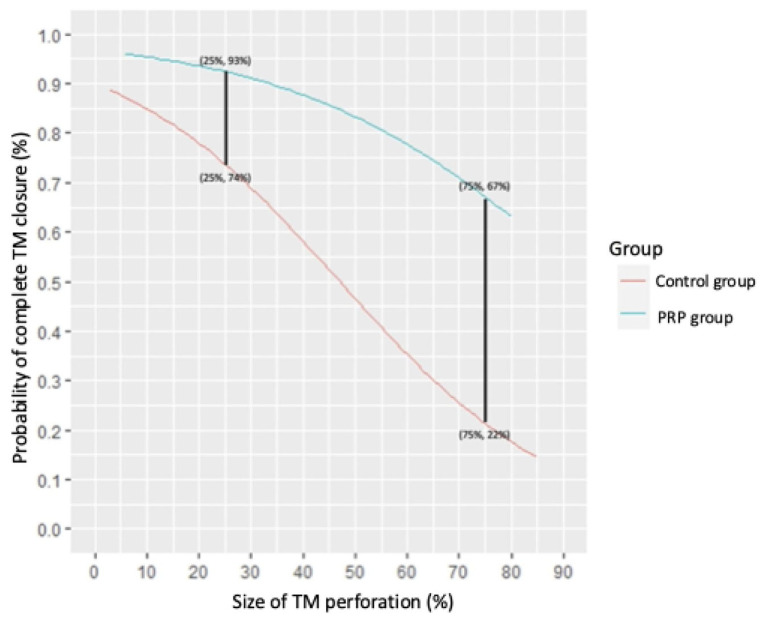
The probability of successful complete tympanic membrane healing in relation to perforation size for both the PRP and control groups. The perforation size is expressed as a percentage of the total tympanic membrane surface area. Legend: PRP—platelet-rich plasma. Note: Two key data points are presented. For perforations covering 25% of the tympanic membrane surface, the success rate of complete healing in the PRP group is 93% compared to 74% in the control group. For perforations covering 75% of the tympanic membrane surface, the success rate in the PRP group drops to 67%, while, in the control group, it is significantly lower, at just 22%. Detailed equations for the calculations are provided in Appendix A.

Our study demonstrated that the use of PRP and PRG significantly enhanced the success rate of tympanic membrane closure following tympanoplasty with temporalis fascia. As illustrated in Figure 4, the curve for the PRP group consistently lies above that of the control group, indicating superior healing outcomes. Notably, the gap between the two groups widens with larger perforation sizes, emphasizing the greater efficacy of PRP in promoting closure of more extensive tympanic membrane defects.

The use of PRP and PRG did not yield statistically significant improvements in hearing outcomes or quality of life, suggesting that, while PRP and PRG may enhance structural healing, their impact on functional recovery and patient-reported outcomes may be limited.

To the best of our knowledge, this is the first study to analyze the simultaneous application of PRP soaked on atelocollagen sponge and PRG placed directly on the tympanic membrane. The main advantage of the study is its prospective, double-blinded, and evenly randomized design, which ensures robust methodology and minimizes bias. Additional objective measures, such as tympanic membrane closure rates and precise perforation size assessments, were employed to enhance accuracy and reliability. Another significant advantage of this study is the selection of PRP as a material to promote tympanic membrane healing. As an autologous product, PRP carries a low risk of adverse reactions and is both cost-effective and easy to prepare on-site. This combination of safety, affordability, and simplicity makes PRP a practical and patient-centered choice for clinical application [22].

The limitations of this study include the relatively small sample size and the short duration of follow-up.

While tympanoplasty has been extensively studied, the potential role of autologous healing stimulators, such as PRP, has received comparatively less attention. However, in recent years, a few prospective randomized controlled trials and systematic reviews have been published [7,8,23,24,25].

Meta-analyses published in 2021 and 2022 support the therapeutic potential of PRP and PRG in enhancing tympanic membrane healing [7,26]. These analyses highlighted that PRP not only increases the likelihood of complete tympanic membrane healing but also reduces the risk of postoperative complications, such as infections [7]. In our study, postoperative infections occurred in four patients, leading to incomplete tympanic membrane closure. Notably, all affected patients were from the control group and did not receive PRP.

However, the meta-analyses did not demonstrate a significant impact of PRP on postoperative hearing improvement [7,26]. These findings align closely with the results observed in our study. Meta-analyses published in 2024 showed that platelet concentrates may improve graft uptake and air-bone gap gain and reduce complications in COM patients undergoing myringoplasty. They advised caution due to the relatively small sample sizes, as well as inconsistent reporting across the included trials [24].

Recently published randomized controlled trials investigating the therapeutic effects of PRP on tympanic membrane regeneration present divided findings [8,23,25,27]. Sharma et al. (2022) [25] and Akash et al. (2023) [23] reported that PRP did not have a statistically significant effect on the tympanic membrane closure rates. Both studies utilized temporalis fascia as the graft material. Sharma et al. (2022) [25] applied PRP by soaking it on atelocollagen sponge; however, their PRP was prepared using a single-spin method, which most probably resulted in a lower concentration of platelets and extracellular vesicles compared to our study [9]. Akash et al. (2023) [23], in contrast, soaked the fascia directly with plasma, using a smaller volume of PRP than the method employed in our study.

Mandour et al. (2023) [8] investigated cartilage with perichondrium compared to fat graft tympanoplasty, with PRP applied laterally. Their findings showed similar success rates between the two groups.

Al-Arman et al. (2024) [27] compared fascia with PRP to cartilage tympanoplasty. Their preparation of PRG differed from ours; while we activated PRG exogenously, they used a blood clot from a tube without anticoagulant, which likely resulted in a lower concentration of regenerative factors.

These variations in preparation methods and application protocols may explain the discrepancies in outcomes across studies and highlight the need for standardized approaches in future research.

The success observed in our study may be attributed to the application of PRG to the outer surface of the reconstructed tympanic membrane, which likely promotes rapid and effective regeneration. This approach may reduce the risk of temporalis fascia migration and enhance the overall success rate of tympanic membrane healing [28].

Most importantly, since PRP did not lead to hearing deterioration, we can conclude that PRP and PRG do not negatively affect inner ear function and are safe for use. PRP’s composition is similar to that of a regular blood clot that forms in wounds and does not promote bacterial growth more than any other clot. Additionally, the pH of PRP (6.5–6.7) is slightly lower than that of a mature blood clot (7.0–7.2), suggesting that PRP may even inhibit bacterial growth [29].

Although our study demonstrated that the use of PRP and PRG significantly improved the success rate of tympanic membrane closure following tympanoplasty with temporalis fascia, comparing the effects of PRP remains inherently challenging due to variability in its preparation and application across different studies. Different techniques for PRP or PRG preparation and their inconsistent delivery to the surgical site complicate the ability to draw reliable conclusions. Furthermore, the use of various graft materials, such as fat, cartilage, or fascia, adds another layer of complexity, making an objective comparison even more difficult.

The existing literature indicates that cartilage grafts are particularly effective in tympanoplasty, achieving high success rates [30]. However, other grafting techniques, such as fascia or perichondrium, appear less effective for the closure of large tympanic membrane perforations. In such cases, as demonstrated by our study, PRP may offer a valuable adjunct to enhance healing outcomes.

Additionally, this raises an important clinical consideration: whether it is more appropriate to use cartilage grafts or to employ PRP as an alternative or adjunctive treatment. PRP could be especially advantageous in less invasive procedures, such as transcanal approaches performed under local anesthesia, where minimizing invasiveness is a priority. Standardized studies comparing these approaches are needed to better define the optimal treatment strategies for various clinical scenarios.

## 5. Conclusions

Our study suggests that the application of platelet-rich plasma (PRP) and platelet-rich gel (PRG) may be associated with improved tympanic membrane closure rates following tympanoplasty with temporalis fascia, particularly in cases involving larger perforations. These autologous, patient-derived preparations are biologically active and may support collagen synthesis, epithelialization, and angiogenesis, without evidence of adverse effects on inner ear function, indicating a favorable safety profile. Although PRP and PRG did not lead to statistically significant improvements in hearing outcomes or quality of life in this cohort, their potential role in enhancing structural healing warrants further investigation. The clinical efficacy of PRP appears to be influenced by preparation protocols and methods of application, which could contribute to the heterogeneity seen in the current literature. Importantly, no postoperative infections were observed in the PRP/PRG groups, supporting prior findings that suggest a possible protective effect. The simultaneous use of PRP on an atelocollagen sponge and PRG on the tympanic membrane surface represents a novel, biologically personalized approach that may facilitate tissue repair in select cases. These findings highlight the potential of autologous platelet-based therapies as adjuncts in minimally invasive tympanoplasty under local anesthesia. However, variability in surgical techniques, graft materials, and PRP formulations underscores the need for standardized protocols and larger, controlled studies to better define their role in individualized otologic care.

## Figures and Tables

**Table 1:** Participant demographics.

	PRP Group (n = 36)	Control Group (n = 36)	*p*-Value
male	19	18	1000 *
female	17	18	
children	3	5	0.71 *
Age			
M ± SD [years]	40.69 ± 18.27	32.42 ± 21.77	0.045 **
Size of tympanic membrane perforation			
Mdn [%]	31	25.5	1000 **

Legend: PRP—platelet-rich plasma; *p*—*p*-value; M—Mean; Mdn—Median; SD—Standard deviation; *—Fisher’s exact test; **—Mann–Whitney *U* test; *p*-value < 0.05 was considered as a statistically significant difference.

**Table 2:** Study outcomes.

	PRP Group (n = 36)	Control Group (n = 36)	*p*-Value
**Complete tympanic membrane closure**	32/36 (88.9%)	24/36 (66.7%)	*p* < 0.05 *
**Pure tone average**			*p* = 0.30 **
*Air conduction Mdn—Pre op.*	39.75	32.50	
*Air conduction Mdn—Post op.*	24.50	18.25	
*Bone conduction Mdn—Post op.*	13.21	10.35	
*Bone conduction Mdn—Pre op.*	13.57	10.35	
**COMQ-12 questionnaire**			*p* = 0.16 ***
*Pre op.*	26.00 ± 10.40	26.02 ± 10.46	
*Post op.*	15.56 ± 9.03	15.60 ± 8.95	

Legend: PRP—platelet-rich plasma; *p*—*p*-value; M—Mean; Mdn—Median; SD—Standard deviation; *—Fisher’s exact test; **—Mann–Whitney *U* test; ***—*t*–test for independent samples; *p*-value < 0.05 was considered as a statistically significant difference.

## Data Availability

The data supporting the findings of this study are stored in a secure local database and are available from the corresponding author upon reasonable request. Access to the data is restricted due to patient privacy and ethical considerations.

## Abbreviations

The following abbreviations are used in this manuscript:

PRP	Platelet-Rich Plasma
PRG	Platelet-Rich Gel
PTA	Pure Tone Audiometry
COMQ-12	Chronic Otitis Media Questionnaire (12-item version)
EAC	External Auditory Canal
ET	Eustachian Tube
ME	Middle Ear
dB	Decibels
M	Mean
Mdn	Median
SD	Standard Deviation
CI	Confidence Interval
RCT	Randomized Controlled Trial
CaCl₂	Calcium Chloride
COM	Chronic Otitis Media
JAMA	Journal of the American Medical Association
CONSORT	Consolidated Standards of Reporting Trials
USA	United States of America
R	R Statistical Software

## Appendix A

### Appendix A.1. Inclusion and Exclusion Criteria

**Table A1:** Criteria for inclusion in the research.

Inclusion Criteria	Exclusion Criteria
dry ear drum perforation dry middle ear cavity absence of exclusion criteria	signs of cholesteatoma anemia thrombocytopenia chronic use of immunomodulating agents and/or antimicrobial drugs the presence or condition after treatment of malignancy in the area of the ear positive current history of systemic infectious disease positive current history of autoimmune disease inability and/or refusal of the patient to participate in the research pregnancy and/or breastfeeding

### Appendix A.2. Equations for Calculating the Probability of Tympanic Membrane Healing

Using logistic regression, we can calculate the probability of tympanic membrane healing based on the size of the perforation in both the PRP group and the control group.

(A1) $$P_{PRP}\;=\;\frac{e^{3.41+\left(-0.04\right)x}}{1+e^{3.41+\left(-0.04\right)x}}$$

(A2) $$P_{control}\;=\;\frac{e^{2.20+\left(-0.05\right)x}}{1+e^{2.20+\left(-0.05\right)x}}$$

where *P_PRP_* is the probability of tympanic membrane healing in the PRP group, *P_control_* is the probability of tympanic membrane healing in the control group, *x* represents the size of the perforation expressed as a fraction (0–1), and *e* is Euler’s number (a mathematical constant; *e* = 2.718).

## References

[1] Mohamad S.H., Khan I., Hussain S.S.M. (2012). Is Cartilage Tympanoplasty More Effective than Fascia Tympanoplasty? A Systematic Review. Otol. Neurotol..

[2] Sheehy J.L., Anderson R.G. (1980). Myringoplasty: A Review of 472 Cases. Ann. Otol. Rhinol. Laryngol..

[3] Balough B.J. (2009). Evaluation of Prognostic Factors and Middle Ear Risk Index in Tympanoplasty. Yearb. Otolaryngol. Head. Neck Surg..

[4] De Seta E., De Seta D., Covelli E., Viccaro M., Filipo R. (2013). Type I Tympanoplasty with Island Chondro-Perichondral Tragal Graft: The Preferred Technique?. J. Laryngol. Otol..

[5] De Seta E., Covelli E., De Seta D., Mancini P., Filipo R. (2010). Cartilage Tympanoplasty: How to Reduce Surgery Time. J. Laryngol. Otol..

[6] Dinaki K., Grigoriadis N., Vizirianakis I.S., Constantinidis J., Triaridis S., Karkos P. (2024). The Impact of Submucosal PRP Injection on Wound Healing after Endoscopic Sinus Surgery: A Randomized Clinical Trial. Eur. Arch. Otorhinolaryngol..

[7] Huang J., Shi Y., Wu L., Lv C., Hu Y., Shen Y. (2021). Comparative Efficacy of Platelet-Rich Plasma Applied in Myringoplasty: A Systematic Review and Meta-Analysis. PLoS One.

[8] Mandour M.F., Elsheikh M.N., Amer M., Elzayat S., Barbara M., Covelli E., Elfarargy H.H., Tomoum M. (2023). The Impact of Adding Platelet-Rich Plasma during Fat Graft Myringoplasty for Managing Medium-Sized Tympanic Membrane Perforations: A Prospective Randomized Case-Control Study. Am. J. Otolaryngol..

[9] Vozel D., Božič D., Jeran M., Jan Z., Pajnič M., Pađen L., Uršič B., Iglič A., Kralj-Iglič V., Battelino S. (2020). Treatment with Platelet- and Extracellular Vesicle-Rich Plasma in Otorhinolaryngology-a Review and Future Perspectives. Advances in Biomembranes and Lipid Self-Assembly.

[10] Yadav S.P.S., Malik J.S., Malik P., Sehgal P.K., Gulia J.S., Ranga R.K. (2018). Studying the Result of Underlay Myringoplasty Using Platelet-Rich Plasma. J. Laryngol. Otol..

[11] Yan C.H., Jang S.S., Lin H.-F.C., Ma Y., Khanwalkar A.R., Thai A., Patel Z.M. (2023). Use of Platelet-Rich Plasma for COVID-19–Related Olfactory Loss: A Randomized Controlled Trial. Int. Forum Allergy Rhinol..

[12] Bielecki T.M., Gazdzik T.S., Arendt J., Szczepanski T., Król W., Wielkoszynski T. (2007). Antibacterial Effect of Autologous Platelet Gel Enriched with Growth Factors and Other Active Substances: An in Vitro Study. J. Bone Jt. Surg. Br..

[13] Tao S.-C., Guo S.-C., Zhang C.-Q. (2017). Platelet-Derived Extracellular Vesicles: An Emerging Therapeutic Approach. Int. J. Biol. Sci..

[14] (2000). World Medical Association Declaration of Helsinki: Ethical Principles for Medical Research Involving Human Subjects. JAMA.

[15] Abramson J.H. (2011). WINPEPI Updated: Computer Programs for Epidemiologists, and Their Teaching Potential. Epidemiol. Perspect. Innov..

[16] Schulz K.F., Altman D.G., Moher D. (2010). CONSORT 2010 Statement: Updated Guidelines for Reporting Parallel Group Randomised Trials. PLoS Med..

[17] Lajdam G.B., Alahmadi R.A., Alhakami M., Ghaddaf A.A., Abdulhamid A.S., Alahmadi A., Abdelsamad Y., Hagr A. (2023). Comparison of Temporalis Muscle Fascia and Cartilage Grafts for Primary Type 1 Tympanoplasty: A Meta-Analysis of Randomized Controlled Trials. Eur. Arch. Otorhinolaryngol..

[18] Nicholas Jungbauer W., Jeong S., Nguyen S.A., Lambert P.R. (2023). Comparing Myringoplasty to Type I Tympanoplasty in Tympanic Membrane Repair: A Systematic Review and Meta-Analysis. Otolaryngol. Head. Neck Surg..

[19] Zwierz A., Haber K., Sinkiewicz A., Kalińczak-Górna P., Tyra J., Mierzwiński J. (2019). The Significance of Selected Prognostic Factors in Pediatric Tympanoplasty. Eur. Arch. Otorhinolaryngol..

[20] Illés K., Gergő D., Keresztély Z., Dembrovszky F., Fehérvári P., Bánvölgyi A., Csupor D., Hegyi P., Horváth T. (2023). Factors Influencing Successful Reconstruction of Tympanic Membrane Perforations: A Systematic Review and Meta-Analysis. Eur. Arch. Otorhinolaryngol..

[21] Xu N., Wang L., Guan J., Tang C., He N., Zhang W., Fu S. (2018). Wound Healing Effects of a Curcuma Zedoaria Polysaccharide with Platelet-Rich Plasma Exosomes Assembled on Chitosan/Silk Hydrogel Sponge in a Diabetic Rat Model. Int. J. Biol. Macromol..

[22] Steiner N., Vozel D., Urbančič J., Božič D., Kralj-Iglič V., Battelino S. (2022). Clinical Implementation of Platelet- and Extracellular Vesicle-Rich Product Preparation Protocols. Tissue Eng. Part. A.

[23] Akash, Datta R., Suri G.S., Mucha S., Sheikh M.A., Taneja N.S. (2023). A Randomised Controlled Trial on the Efficacy of Topical Application of Autologous Platelet Rich Plasma (PRP) on Graft Uptake Rate in Adults Undergoing Type 1 Tympanoplasty for Inactive COM Mucosal Disease. Indian. J. Otolaryngol. Head. Neck Surg..

[24] Alahmadi R.A., Lajdam G.B., Aghashami A., Hamdan D., Almalki A.H., Altalhi A.A., Amoodi H.A. (2025). Platelet Concentrates Impact on Myringoplasty Outcomes in Chronic Otitis Media Patients: Systematic Review and Meta-Analysis. Otolaryngol. Head. Neck Surg..

[25] Sharma P., Parida P.K., Preetam C., Mukherjee S., Nayak A., Pradhan P. (2022). Outcome of Temporalis Fascia Myringoplasty With and Without Use of Platelet Rich Plasma: A Randomized Control Trial. Indian. J. Otolaryngol. Head. Neck Surg..

[26] Huang J., Teh B.M., Zhou C., Shi Y., Shen Y. (2022). Tympanic Membrane Regeneration Using Platelet-Rich Fibrin: A Systematic Review and Meta-Analysis. Eur. Arch. Otorhinolaryngol..

[27] Al-Arman A.M., Moneir W., Amer H.E., Ebada H.A. (2024). Platelet Rich Fibrin Augmented Tympanoplasty versus Cartilage Tympanoplasty: A Randomized Clinical Trial. Eur. Arch. Otorhinolaryngol..

[28] Navarrete Álvaro M.L., Ortiz N., Rodriguez L., Boemo R., Fuentes J.F., Mateo A., Ortiz P. (2011). Pilot Study on the Efficiency of the Biostimulation with Autologous Plasma Rich in Platelet Growth Factors in Otorhinolaryngology: Otologic Surgery (Tympanoplasty Type I). ISRN Surg..

[29] Choi J., Minn K.W., Chang H. (2012). The Efficacy and Safety of Platelet-Rich Plasma and Adipose-Derived Stem Cells: An Update. Arch. Plast. Surg..

[30] Jain A., Samdani S., Sharma M.P., Meena V. (2018). Island Cartilage vs Temporalis Fascia in Type 1 Tympanoplasty: A Prospective Study. Acta Otorrinolaringol. Esp. (Engl. Ed.).

